# Decreased severity of collagen antibody and lipopolysaccharide-induced arthritis in human IL-32β overexpressed transgenic mice

**DOI:** 10.18632/oncotarget.6160

**Published:** 2015-10-19

**Authors:** Mi Hee Park, Do-Young Yoon, Jung Ok Ban, Dae Hwan Kim, Dong Hun Lee, Sukgil Song, Youngsoo Kim, Sang-Bae Han, Hee Pom Lee, Jin Tae Hong

**Affiliations:** ^1^ College of Pharmacy and Medical Research Center, Chungbuk National University, Osongsaengmyeong 1-ro, Osong-eup, Cheong-ju, Chungbuk, Republic of Korea; ^2^ Department of Bioscience and Biotechnology, Laboratory of Cell Biology and Immunobiochemistry, Bio/Molecular Informatics Center, Konkuk University, Hwayang-dong 1, Gwangjin-gu, Seoul, Republic of Korea; ^3^ Osong Medical Innovation Foundation, Osongsaengmyeong 1-ro, Osong-eup, Cheongwon-gun, Chungbuk, Republic of Korea

**Keywords:** IL-32β, anti-arthritis, anti-inflammatory cytokine, Immunology and Microbiology Section, Immune response, Immunity

## Abstract

Interleukin (IL)-32, mainly produced by T-lymphocytes, natural killer cells, epithelial cells, and blood monocytes, is dominantly known as a pro-inflammatory cytokine. However, the role of IL-32 on inflammatory disease has been doubtful according to diverse conflicting results. This study was designed to examine the role of IL-32β on the development of collagen antibody (CAIA) and lipopolysaccharide (LPS)-induced inflammatory arthritis. Our data showed that the paw swelling volume and clinical score were significantly reduced in the CAIA and LPS-treated IL-32β transgenic mice compared with non-transgenic mice. The populations of cytotoxic T, NK and dendritic cells was inhibited and NF-κB and STAT3 activities were significantly lowered in the CAIA and LPS-treated IL-32β transgenic mice. The expression of pro-inflammatory proteins was prevented in the paw tissues of CAIA and LPS-treated IL-32β transgenic mice. In addition, IL-32β altered several cytokine levels in the blood, spleen and paw joint. Our data indicates that IL-32β comprehensively inhibits the inflammation responses in the CAIA and LPS-induced inflammatory arthritis model.

## INTRODUCTION

IL-32, formerly known as natural killer cell transcript 4, is produced by T cells, epithelial cells, monocytes, NK cells, and fibroblasts after being stimulated by IL-2, IL-12, IL-18, and interferon-gamma (IFN-γ) [1, 2]. Mouse homologues of IL-32 have not yet been reported, however, IL-32 has several splice variants such as IL-32α, IL-32β, IL-32γ, IL-32δ, IL-32ε, and IL-32τ. Among these, IL-32α has the shortest transcription (134 amino acids) and 32β is a dominant variant (188 amino acids), whereas IL-32γ isoform (168 amino acids) has the strongest biological activity [2, 3]. Two other isoforms, IL-32ε (179 amino acids) and τ, were recently identified, but these isoforms are not ubiquitously expressed [4]. A recent study shows that IL-32γ, which occurs naturally, can be spliced into a less potent IL-32β [5]. IL-32γ has shown anti-viral and anti-mycobacterial activities. IL-32β and IL-32γ are associated with cancer cell growth and development of inflammatory diseases including RA. However, IL-32α, may not be correlated with inflammation [6].

IL-32 exhibits pro-inflammatory properties and induces other chemokines and pro-inflammatory cytokines such as tumor necrosis factor-alpha (TNF-α), IL-1, IL-6, and IL-8. Due to such pro-inflammatory properties, IL-32 is considered to develop the various inflammatory diseases, including inflammatory arthritis [5], inflammatory bowel disease [7], and certain tumors [8]. Although the receptor and analogue of IL-32 have not yet been identified in mice, human IL-32 exerts pro-inflammatory effects as an inducer of inflammatory arthritis [9, 11]. IL-32 is considered pro-inflammatory because it induces other pro-inflammatory cytokines and chemokines such as TNF-α, IL-1β, IL-6 and IL-8 by activation of NF-κB and p38 MAPK and because elevated levels of IL32 were notable in synovial tissues of patients with rheumatoid arthritis where those levels correspond to the severity of diseases [5, 9, 12]. It has been reported that IL-32 enhanced the susceptibility to lipopolysaccharide-induced arthritis through the induction of TNFα in IL-32α transgenic mice [13]. IL-32γ provokes cellular infiltration of inflammatory cells and cartilage damage in the joint spaces of recombinant human IL-32γ administrated mice [10]. Therefore, existing studies up to now on the function of expressed IL-32 *in vivo* have focused on the induction of other pro-inflammatory cytokines such as TNF-α, IL-1, and IL-6 which are considered clinically causative in the development of inflammatory arthritis.

Despite that the evidence in a number of previous studies shows that IL-32 is a pro-inflammatory cytokine in the development of inflammatory arthritis, various efforts have also been reported with opposite results by demonstrating the inhibitory effects on inflammation responses. Transgenic mice expressing human IL-32γ initially exhibited greater inflammation in an induced colitis model compared to wild type mice; as the disease progressed, the transgenic mice recovered and healed more rapidly than did the wild type mice [14]. It has also been observed that the splicing of IL-32γ into IL-32β contributes to reduced chronic inflammation causing arthritis [5]. Another relevant result also found was that the production of pro-inflammatory cytokines and tumor growth were inhibited in IL-32γ over-expressed transgenic mice inoculated with melanoma [8]. Moreover, IL-32β increased the anti-inflammatory cytokine IL-10 level in human cell lines [15]. It is therefore necessary to define more comprehensive properties of IL-32 in the chronic inflammatory response. We chose IL-32β for our experiment because of its possible anti-inflammatory properties in certain diseases, as well as the most biological active IL-32γ can be spliced into IL-32β contributing to reduced chronic inflammation [5] as well as being the most biologically active IL-32γ that can be spliced into IL-32β, contributing to reduced chronic inflammation. Thus, we investigated the role of IL-32β in the development of inflammatory arthritis using IL-32β over-expressed transgenic mice.

## RESULTS

### Generation of IL-32β transgenic mice, and the expression of IL-32β in the mice

To investigate the role of IL-32β in the development of inflammatory arthritis *in vivo*, we generated transgenic mice by inserting a human IL-32β gene with a chicken beta-actin promoter to create an over-expressing expression of human IL-32β. Prior to generating the IL-32β transgenic mice, we confirmed that the IL-32β cDNA was properly translated into the IL-32 protein using GST-fused IL-32β protein expression in *Escherichia coli*. The GST-fused IL-32β protein was ascertained by Western blotting with an anti-IL-32 reactive monoclonal antibody KU32-52, as described elsewhere [15, 16]. RT-PCR and Western blotting analysis revealed that hIL-32β was expressed in paw tissue (Supplementary Figure 1). IL-32 was highly expressed in the heart, liver, thymus, and intestine, whereas expression of hIL-32β in the tissues of the non-transgenic mice was not found (data not shown). The IL-32 level in the sera of IL-32β transgenic mice (1.7±0.2 ng/ml), detected by the ELISA method, was increased over the wild type of mice (0.2±0.04 ng/ml).

### Inhibition of arthritis by IL-32β

**Figure 1:**
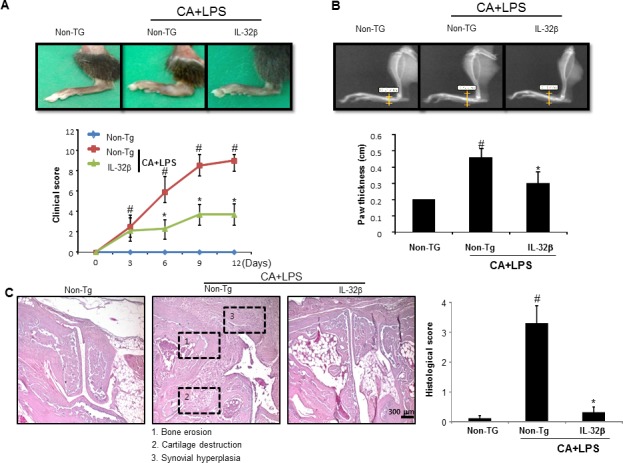
Effect of IL32β in inflammatory arthritis Anti-collagen II mAbs (CA) was injected on day 0, followed by 50 μg of LPS on day 3. CA+LPS-induced mouse was evaluated on arthritis severity on day 12. Representative photographs of joint swelling of the hind paws in mice (*n* = 10) (**A.** upper panel). Clinical score of a total of 10 mice for each pair were checked from 0 to 4 as described in the Methods (**A.** lower panel). A radiographic examination of hind paws revealed tissue swelling at the paw after 12 days (**B.** upper panel). Paw swellings of soft tissues were radiologically evaluated by taking soft x-ray photographs (*n* = 10) (**B.** lower panel). Representative histopathologies of the ankle joints stained with hematoxylin and eosin (H&E) (**C.** left panel). Histological examinations of a total of 5 mice for each pair were checked from 0 to 4 as described in Material (**C.** right panel). ^#^*P* < 0.05 compared non transgenic (Non-Tg) mice without CAIA and LPS. *P < 0.05 compared with Non-Tg mice treated with CAIA and LPS.

We intraperitoneally administered 5 mg/mouse of (anti-Collagen) mAB cocktail on day 0 and treated it with 50 μg/mouse of lipopolysaccharide (LPS) that was intraperitoneally administered as well as on day 3. Until day 3, there was not much difference in the induction of arthritis between non transgenic and IL-32β overexpressed transgenic mice in a collagen antibody and LPS-induced arthritis model. However, after day 4, hind paw edema was significantly increased in the non-transgenic mice, but reduced in the IL-32β transgenic mice (Figure 1A). Consistent with the inhibitory effect on the paw swelling volume, the clinical score was also reduced in IL-32β transgenic mice (Figure 1B). A result of a radiographic examination of the hind paws signified fibrosis, synoviocyte hyperplasia, bone erosion, and cartilage destruction at the joint margin of the 12 days post-injected non-transgenic mice (Figure 1C), whereas the damage was markedly reduced in the IL-32β transgenic mice (Figure 1C). However, the CAIA and LPS treatment did not decreased the body weight and did not caused behavioral alternation (data not shown).

### Reduction of immune responses by IL-32β

**Figure 2:**
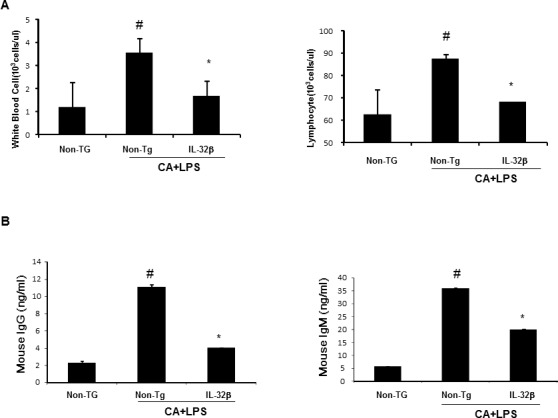
Effect of IL32β on immune responses in inflammatory arthritis IL32β mice In CAIA with LPS-induced Non-Tg and IL32β Tg mouse, the number of WBC and lymphocyte was counted in blood **A.** Immunoglobulin G and M levels were detected as described in Methods **B.** Value is presented as mean ± SD from 10 mice. ^#^*P* < 0.05 compared Non-Tg mice without CAIA and LPS. *P < 0.05 compared with Non-Tg mice treated with CAIA and LPS.

**Figure 3:**
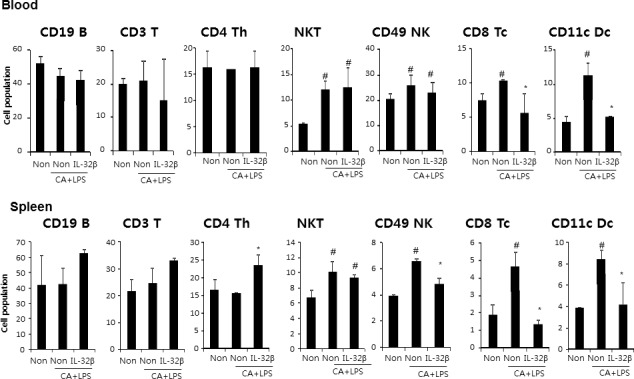
Effect of IL32β on immune cells in inflammatory arthritis IL32β mice The cell number of CD19B, CD3 T, CD4 Th, NKT, CD49 NK, CD8 Tc, and CD11c Dc were detected in blood **A.** and spleen **B.** Value is presented as mean ± SD from 10 mice. ^#^*P* < 0.05 compared Non-Tg mice without CAIA and LPS. *P < 0.05 compared with Non-Tg mice treated with CAIA and LPS. The experiments shown in Fig. 3 were repeated in triplicate with similar results.

**Figure 4:**
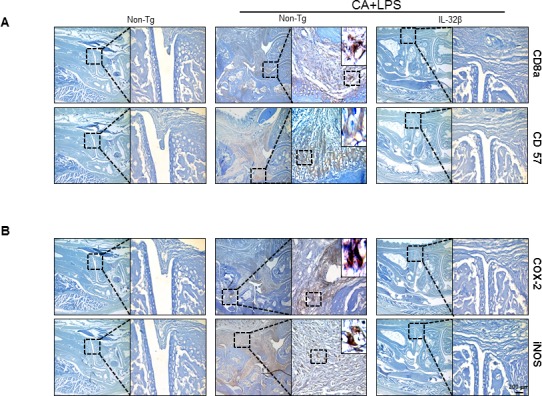
Effect of IL32β on inflammatory cells and expression of inflammatory genes in inflammatory arthritis IL32β mice The expression level of CD8a (a cytotoxic T cell), CD57 (for NK cells), and COX2/iNOS (inflammatory markers) in CAIA with LPS-treated IL-32β transgenic and Non-Tg mice were determined with immunohistochemical analysis as described in Methods. The experiments shown in Figure 4 were repeated in triplicate with similar results.

When an inflammation response is induced, innate immune cells like WBC and lymphocytes should flock to sites near the inflammatory regions. The number of WBC and lymphocytes increased in the blood of CAIA and LPS-treated non transgenic mice but significantly lower in the blood of CAIA and LPS-treated IL-32β transgenic mice (Figure 2A). Associated with the reduced number of WBC and lymphocytes, the immunoglobulin(Ig) G and M levels were also reduced in the blood of CAIA and LPS-treated IL-32β transgenic mice (Figure 2B). The lymph nodes were significantly enlarged (Supplementary Figure 2A) in the CAIA and LPS treated non-transgenic mice; but reduced in the CAIA and LPS-treated IL-32β transgenic mice. Also, the CD8a positive cytotoxic T cells and F4/80 positive macrophage cells are increased in the CAIA and LPS treated non-transgenic mice; but reduced in the CAIA and LPS-treated IL-32β transgenic mice (Supplementary Figure 2B and 2C). Moreover, NO generation in the IL-2 treated spleen of the CAIA and LPS-treated non-transgenic mice was noticeably elevated, but reduced in the CAIA and LPS-treated IL-32β transgenic mice (Supplementary Figure 3). The involvement of acquired immune cells in the blood, spleen, inguinal lymph nodes (LNs) and paw joint was compared between the CAIA and LPS-treated non transgenic mice and the CAIA and LPS-treated IL-32β transgenic mice. As shown in Figure 3, in the blood and spleen, the number of total B and T cells was not different, but the number of CD4 positive helper T cells was significantly elevated in the spleen of the CAIA and LPS-treated IL-32β transgenic mice. NKT cells were elevated by the treatment of the CAIAs and LPS in both the blood and spleen. However, cytotoxic T cells, NK cells as well as dendritic cells were elevated in the CAIA and LPS-treated non-transgenic mice, but the number of these cells was significantly reduced in the spleen and the number of cytotoxic T cells and dendritic cells was significantly reduced in the blood of CAIA and LPS-treated IL-32β transgenic mice (Figure 3). In the paw joint and lymph node of the CAIA and LPS-treated non-transgenic mice, the number of CD8a positive cytotoxic T cells, CD57 positive NK cells were significantly elevated, but were reduced in the paw joint and lymph node of the CAIA and LPS-treated IL-32β transgenic mice (Figure 4A).

### IL-32β decreases NF-κB and STAT3 activity in paw joint tissues

**Figure 5:**
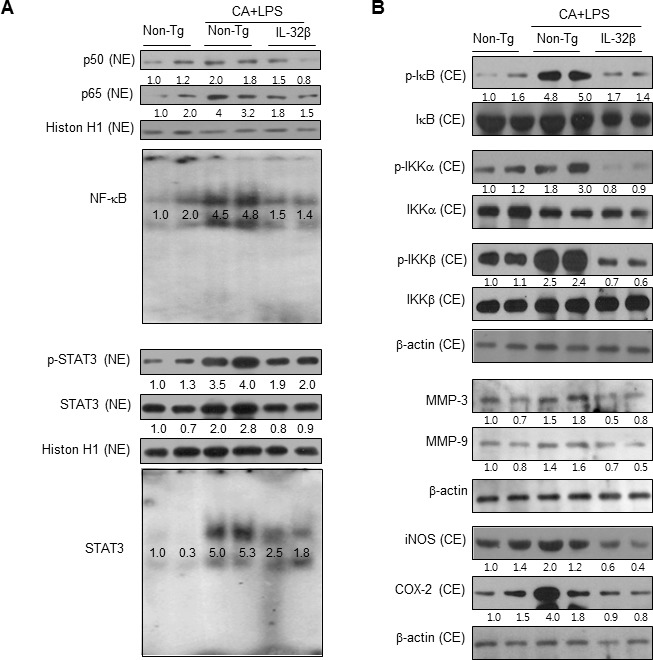
Effect of IL32β on the activity of NF-κB and STAT3 in the paw joints of inflammatory arthritis IL32β mice **A.** CAIA with LPS treated mice joint tissues (two animals) were lysated and detected with antibodies against p50, p65, Histion H1, p-STAT3, and STAT3 using western-blotting. The DNA-binding activity of NF-κB and STAT3 was detected by EMSA **B.** IKK, p-IKK, IκB, p-IκB, MMP-3, MMP-9, iNOS, and COX-2 were detected using specific antibodies. The experiments shown in Fig. 5 were repeated with another three set of two animals. The results were similar.

The activation of NF-κB stimulates inflammation and development of arthritis. To determine whether IL-32β inhibits the activation of NF-κB in paw joints, we first specified the DNA binding activity of NF-κB by EMSA, and translocation of p65 and p50 into the nucleus and IκBα protein degradation as well as phosphorylation of IKKα and IKKβ by Western blot analysis. The DNA binding activity of NF-κB and the translocation of p65 and p50 into the nucleus and IκBα protein degradation as well as p-IKKα and p-IKKβ were significantly increased in the paw joint of CAIA and LPS-treated non transgenic mice, but these levels were significantly decreased in the paw joint of the CAIA and LPS-treated IL-32β transgenic mice (Figure 5B). STAT3 is also implicated in inflammatory-associated arthritis and in maintaining of the constitutive activation of NF-κB. Thus, we investigated whether STAT3 activation was also inhibited in CAIA and LPS-treated IL-32β transgenic mice. Consistent with the inhibitory effect on NF-κB activation, STAT3 phosphorylation as well as DNA binding activity were significantly lowered in the paw joint tissues of the CAIA and LPS-treated IL-32β transgenic mice compared to those in the paw joint tissues of non transgenic mice (Figure 5A).

### IL-32β inhibits the expression of inflammatory proteins in paw joint tissues

We also investigated whether IL-32β inhibited inflammatory gene expression by the inhibition of NF-κB and STAT3 signals in the paw joint, and thus suppresses the development of arthritis. The levels of the inflammatory protein expression such as iNOS, COX-2, MMP-3 and MMP-9 were significantly increased in the paw joint tissues of the CAIA and LPS-treated non-transgenic mice, but the expression levels were significantly prevented in the CAIA and non-treated IL-32β transgenic mice as determined by immunohistochemical analysis (Figure 4B) and Western blotting (Figure 5B).

### IL-32β changes the cytokine levels in the blood, spleen and paw joint tissues

**Figure 6:**
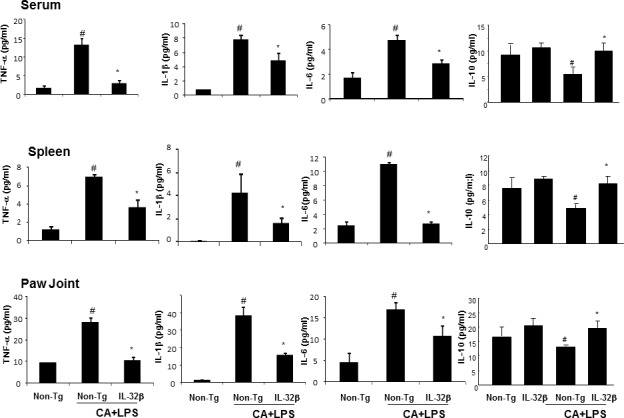
Effect of IL32β on the cytokine levels in the serum, spleen and paw joints of inflammatory arthritis IL32β mice The level of TNF-α, IL-1β, IL-6, and IL-10 were measured by means of ELISA in serum **A.**, spleen **B.**, and paw joint **C.** Value is presented as mean ± SD from 10 mice. ^#^*P* < 0.05 compared Non-Tg mice without CAIA and LPS. *P < 0.05 compared with Non-Tg mice treated with CAIA and LPS.

To investigate whether IL-32β alters other cytokine levels in the blood and immune organ (spleen), and whether the paw joint thus suppresses the development of arthritis, we analyzed cytokine levels by ELISA. The levels of the pro-inflammatory cytokines, such as TNF-α, IL-6 and IL-1, were significantly increased, whereas the level of IL-10, an anti-inflammatory cytokine was significantly decreased in the blood, spleen, and paw joints of CAIA and LPS-treated non-transgenic mice. However, those changes were significantly prevented in CAIA and LPS-treated IL-32β transgenic mice (Figure 6).

## DISCUSSION

In this study, we investigated the role of IL-32β in the development of inflammatory arthritis using IL-32β over-expressed transgenic mice. It was recently reported in a cohort study of a patient suffering from inflammatory arthritis that IL-32 was significantly increased in the synovial tissue and its levels were strongly correlated with the severity of the disease [12, 19]. Moreover, bone marrow transplantation-human IL-32β mice showed exacerbation of collagen-antibody-induced arthritis [9], and recombinant human IL-32γ provoked cellular infiltration of inflammatory cells and cartilage damages in the joint spaces of mice [10]. However, contrast to findings, our results demonstrated that over-expression of IL-32β ameliorated CAIA and LPS induced inflammatory arthritis in IL-32β overexpressed transgenic mice.

It has been observed that the expression of inflammatory cytokines and the infiltration of inflammatory or immune cells into the joint were associated with inflammatory rheumatic arthritis [13, 19]. In this study, we showed that the levels of TNF-α, IL-6 and IL-1β were significantly increased, whereas the level of IL-10 was significantly decreased in the serum, spleen, and paw joints of CAIA and LPS-treated non-transgenic mice, however, those changes were significantly prevented in CAIA and LPS-treated IL-32β transgenic mice. In inflammatory arthritis, IL-1β and TNF-α are strong inflammatory cytokines that are produced mainly from macrophages, and are the two important factors in joint inflammation and bone destruction [20, 21]. IL-6, a known downstream cytokine of IL-1β, is produced from macrophages, activated T cells, B cells and keratinocytes, and stimulates the cells to secrete immunoglobulin and other pro-inflammatory cytokines contributing to the development of inflammatory arthritis [13, 22]. However, IL-10 suppresses the production of TNF-α, IL-1β, IFN-γ, and IL-6, and inhibits the development of inflammatory arthritis [23, 24]. So, our result indicated that IL-32β eventually ameliorated the development of inflammatory arthritis through regulation of several cytokines.

Actually, inflammatory arthritis activates synovial inflammation result from cellular infiltration. The cellular infiltrate includes granulocytes, monocytes/macrophages, NK cells and T cells, all leading to the production of large amounts of chemokines and proinflammatory cytokines [25–27]. Arthritis in B6 IL-10(−/−) mice has been associated with elevated numbers of T cells, NK cells, and NKT cells as well as macrophage infiltration of the infected joint [28]. In agreement with these findings, our data also showed that NKT, NK cells, cytotoxic T and dendritic cells were elevated with the treatment of CAIA and LPS in non transgenic mice, but these elevated cell numbers were significantly decreased in the blood and spleen of IL-32β overexpressed transgenic mice. Immunohistochemical analysis of paw joint also showed activation of the NK cells and cytotoxic T cells in CAIA and LPS treated non-transgenic mice, but not in IL-32β overexpressed transgenic mice. These findings indicate that IL-32β ameliorated CAIA and LPS induced inflammatory arthritis through inhibiting pro-inflammatory cytokine production as well as decrease the infiltration of cytotoxic T cells and NK cells. Actually, we used the acute inflammation system, and it seems that IL-32β suppresses the innate immune system in the inflammatory arthritis. This system, we showed that the activation of NKT, NK cells and dendritic cells that are important for the innate immune system by CAIA and LPS was reduced by IL-32β.

When the paw joint tissues were overexpressed with IL-32β, the expression of genes activated by NF-κB and STAT3, especially inflammatory associated genes, such as iNOS and COX-2 and the DNA binding activity of NF-κB and STAT3 were significantly inhibited. NF-κB and STAT family signals have been recognized as the major pathways responsible for their abilities to lead to the expression of a big array of inflammatory mediators and their roles as the central part of transcription factors in several immune responses [29, 30]. It has been reported that IL-6 induces the activation of NF-κB and STAT3 in the development of inflammatory arthritis [23]. In contrast, IL-10 blocks NF-κB activity and induces the reduction of joint inflammation [24]. Our previous studies also showed that IL-32 inhibited NF-κB and STAT3 in tumor growth models [8, 31] as well as acetoaminophen-induced liver injury and 1-methyl-4-phenyl-1,2,3,6-tetrahydropyridine induced Parkinson models (data not shown). Hence, it is possible that the suppression of the NF-кB and STAT3 signals by IL-32β could correlate with the inhibitory effect of IL-32β on the development of inflammatory arthritis through the reduction of the production of proinflammatory cytokines (TNF-α, IL-1, and IL-6) and the increase of the production of an anti-inflammatory cytokine (IL-10). It was also reported that LPS-induced arthritis was enhanced in IL-32α overexpressed transgenic mice [13]. They showed that the LPS induced development of severe synovitis with substantial articular cartilage degradation in knees of the IL-32α-tg mice. They found that overexpressed IL-32α accelerated production of TNFα upon stimulation with LPS in cultured bone marrow cells derived from the Tg mice. It was also reported that IL-32 protected against *Mycobacterium tuberculosis* infection, and viral infection in differentiated THP-1 human macrophages [32, 33]. IL-32 also suppresses proangiogenic signals in bronchial epithelial cells [34], and promotes the release of IL-4 and IFN-γ inhibitors of osteoclast formation in peripheral blood mononuclear cells [35]. IL-32 is thus considered to represent an anti-inflammatory cytokine.

Even though IL-32 was expressed and the level could be elevated in inflammatory diseases, the role of IL-32 is not clear, whether they act as a preventive or causative factor. As a result, IL-32 could be considered to represent a cytokine to possess contradictory properties as a pro-inflammatory or an anti-inflammatory cytokine according to the different phase, status, and unknown factors in the diseases. Thus, the time and disease status dependences and complicated regulation of IL-32 on the inflammatory responses during the course of inflammatory diseases should be further elucidated. However, our present data suggests that IL-32β could act as a suppressing property in the development of inflammatory arthritis.

## MATERIALS AND METHODS

### Ethics statement

The experimental treatments were carried out according to the guidelines on animal experiments set forth by the Faculty of Disease Animal Model Research Center, Korea Research Institute of Bioscience and Biotechnology (Daejeon, Korea). The protocol was approved and carried out by the Committee of Chungbuk National University, Korea (CBNUA-436-12-02). Surgery was performed under anesthesia by diethyl ether with all efforts to minimize suffering.

### Generation of IL-32β transgenic mice

To generate transgenic mice that expresses hIL-32β, concentrated hIL-32β cDNA was generated as previously described by Oh et al. for IL-32γ transgenic mice [8]. The pCAGGS/hIL-32β plasmid was prepared with the Qiagen MIDI-Prep Kit. To generate IL-32β transgenic mice, a 705-base pair fragment of the hIL-32β gene was sub-cloned into the EcoRI sites of the pCAGGs expression vector containing chicken beta-actin promoter. Prior to generate IL-32β transgenic mice (C57BL6/J background), we confirmed that IL-32β cDNA was properly translated into IL-32 protein using GST-fused IL-32β protein expression in *Escherichia coli*. The GST-fused IL-32β protein was distinguished by Western blotting with an anti-IL-32 reactive monoclonal antibody KU32-52, as described elsewhere [7, 13]. IL-32β insertion was confirmed by amplification of genomic DNA isolated from the transgenic mouse tails using Super Taq PLUS Pre-mix (RexGeneBioTech, Korea) as similar to previous report [7]. IL-32β protein, expressed in the tissues of transgenic mice, was ascertained by RT-PCR and Western blotting with an anti-IL-32 monoclonal antibody KU32-52 [13].

### Induction of inflammatory arthritis in IL-32β transgenic mice

To induce inflammatory arthritis, 5 mg of an anti-collagen II mAbs (CII-Ab, Arthrogen-CIA Arthritogenic Monoclonal Antibody, # 53010: Chondrex, Inc., WA, USA) was injected into 12-week-old male mice (28-30 g weight) in IL-32β on day 0 and followed by 50 μg of LPS (serotype O55:B5, Sigma, St. Louis, MO, USA) on day 3, both by intraperitoneal (i.p.) injection. To evaluate arthritis severity, the following scoring system was employed: 0, no evidence of erythema or swelling: 1, erythema and mild swelling confined to the midfoot (tarsals) or ankle joint: 2, erythema and mild swelling extending from the ankle to the midfoot: 3, erythema and moderate swelling extending from the ankle to the metatarsal joints: 4, erythema and severe swelling encompassing the ankle, foot and digits. The combined limbs total score was recorded each day (maximum score 16).

### Electromobility shift assay (EMSA)

Gel shift assays were performed with an assay kit (Promega, Madison, WI) according to the recommendations of the manufacturer. Briefly, paw joint tissues were washed twice with 1× PBS, followed by the addition of 1 ml of PBS, and then cells were transferred into cold Eppendorf tubes. The cells were spun down at 13,000 rpm for 5 min, and the resulting supernatant was removed. Tissues were suspended in 400 μl of solution A containing 10 mM HEPES, pH 7.9, 1.5 mM MgCl2, 10 mM KCl, 0.5 mM dithiothreitol, 0.2 mM phenylmethylsulfonyl fluoride, and vigorously vortexed. Then, the tissues were allowed to incubate on ice for 10 min and centrifuged at 12,000 rpm for 6 min. The nuclei pellet was resuspended in solution C (solution A + 420 mM NaCl, 20% glycerol) and was allowed to incubate on ice for 20 min. The tissues were centrifuged at 15,000 rpm for 15 minutes, and the final nuclear extract supernatant was collected in a chilled Eppendorf tube. Consensus oligonucleotides for NF-κB (5′-GGGACTTTCC-3′) and STAT3 (5′-TTCTGGGAA-3′) were end-labeled using T4 polynucleotide kinase and [γ-32P] ATP for 10 min at 37°C. Gel shift reactions were assembled and allowed to incubate at room temperature for 10 min followed by the addition of 1 μl (50,000-200,000 cpm) of ^32^P end-labeled oligonucleotide and another 20 min of incubation at room temperature. Subsequently, 1 μl of gel loading buffer addition was made to each reaction and loaded onto a 6% nondenaturing gel and electrophoresis was performed until the dye was four fifths of the way down the gel. Gels were dried at 80°C for 1 hr and exposed to film overnight at −70°C.

### Western blot analysis

Western blot analysis was done as described previously [8]. The membrane was incubated with specific antibodies: mouse monoclonal antibodies against MMP9, p-p65, p65, p-IκB, STAT3, p-STAT3, histone H1 and β-actin (1:500 dilution, Santa Cruz Biotechnology Inc. Santa Cruz, CA, USA), Goat rabbit polyclonal MMP3 (1:500 dilution, Santa Cruz Biotechnology Inc. Santa Cruz, CA, USA), rabbit polyclonal for p50, p-IKKα, IKKα, p-IKKβ, IKKβ, p-IκB and IκB (1:500 dilution, Santa Cruz Biotechnology Inc.) and iNOS and COX-2 (1:1000 dilution, Cayman Chemical, Ann Arbor, Mich, USA). The relative density of the protein bands was scanned by densitometry using MyImage (SLB, Seoul, Korea) and quantified by Labworks 4.0 software (UVP, Inc., Upland, CA, USA).

### Histological techniques

A histological test was performed using the paraffin sections of paw joint tissues obtained from each mouse (*n* = 10 each) on day 9. For histological processing, paws were fixed in phosphate buffer containing 10% formaldehyde and decalcified with 10 % EDTA for 7 days. Paws were processed by routine methods to paraffin blocks. Specimens were sectioned at 6 μm thick and stained with hematoxylin and eosin (H&E). All sections were evaluated histologically by two independent observers. The gradation of arthritis was scored from 0 to 4 according to the intensity of lining layer hyperplasia, mononuclear cell infiltration, and pannus formation, as described previously [17, 18] - 0: normal ankle joint; 1: normal synovium with occasional mononuclear cells; 2: definite arthritis, a few layers of flat to rounded synovial lining cells and scattered mononuclear cells; 3: clear hyperplasia of the synovium with three or more layers of loosely arranged lining cells and dense infiltration with mononuclear cells; 4: severe synovitis with pannus and erosions of articular cartilage and subchondral bone.

### Radiological assessment

Prior to sacrificing the mice, the joints of the hind limb were imaged using an X-ray apparatus (REX-525R, Korea) and industrial X-ray film (Kodak Photo Film, USA) to assess joint damage. The X-ray apparatus was operated at 220 V with a 40 V peak, 0.05 s exposure time, and a 100 cm tube-to-film distance for anterior-posterior projection.

### IgM and IgG detection

The serum levels of IgG and IgM were evaluated with an ELISA kit (Life Diagnostics, USA) according to the manufacturer's protocol. Optical density was measured in an automated microplate reader (Sunrise TM, Tecan, Switzerland).

### Immunohistochemical assay

Immunohistochemical evaluation of COX-2, iNOS, CD57, CD8a, and CD11c3 Paraffin-embedded Immunohistochemistry was performed using the avidin-biotin-peroxidase method. Sections were stained with anti COX-2 antibody (Cayman Chemical, Ann Arbor, MI), iNOS(Cayman Chemical, Ann Arbor, MI), CD8a(Santa Cruz Biotechnology Inc. Santa Cruz, CA ), CD57 (Cayman Chemical, Ann Arbor, MI), and Anti-CD11c hamster monoclonal antibody (for dendritic cell, 1:200, Abcam). Following deparaffinization through a series of graded alcohols and xylene, heat-induced epitope retrieval were completed for all slides by incubating the slides in citrate buffer, pH 6.0. Slides were heated for 5 min in a microwave oven at 50% power for 2 cycles. After microwaving, the slides were placed to cool for 15 min, and then rinsed in distilled water. All incubations were carried out at room temperature, and blocked for endogenous peroxidase activity with 3% H_2_O_2_ for 15 min. Slides were blocked in 5% normal serum (Vector Laboratories, Burlingame, CA) and then incubated with the primary antibody at a dilution of 1:200 for 2 hrs; incubated with the secondary antibody (Vector Laboratories, Burlingame, CA) for 30 min, washed again, and labeled with an avidin-biotin complex (Vector Elite Kit, Vector) for 30 min. Slides were rinsed in water, counterstained with Harris hematoxylin (Harelco, Gibbstown, NJ), and coverslipped with Permount (Surgipath, Richmond, IL).

### Reverse transcription-polymerase chain reaction (RT-PCR)

Total RNA in the joint tissues was extracted by RNeasy (Qiagen, Valencia, CA, USA). The RT reaction was performed using RNA (10 μg) to cDNA Kit (final 50 μl reaction) (Applied Biosystems, USA). The PCR reaction was performed with cDNA (2 μl) as a template using the primers below after an initial 1-min denaturation at 96°C, followed by the indicated cycles of 96°C for 1 min, 60°C or 63°C for 1 min and 72°C for 1 min. The PCR primers used were 5`-ATGTGCTTCCCGAAGGTCCTC-3` and 5`-TCATTTTGAGGATTGGGGTTC-3` for the primer of IL-32, 5`-ACCAAGTGCCACAAAGGAAC-3` and 5`-CTGCAATTGAAGCACTGGAA-3` for the primer of mouse glyceraldehyde-3-phosphate dehydrogenase(GAPDH) respectively

### Enzyme-linked immunosorbent assay (ELISA)

The cytokine (TNF-α, IL-1β, IL-6 and IL-10) levels were evaluated with an ELISA kit (Life Diagnostics, USA) according to the manufacturer's protocol. Briefly, 100 μl of standard and diluted samples in duplicated wells were incubated at room temperature for 45 minutes. After washing the wells 5 times with 1x wash solution, 100 μl of enzyme conjugate reagent was added into each well, then incubated for another 45 minutes. The reaction was stopped by adding 100 μl of stop solution, and the formed color was assayed with a microtiter plate reader (SunriseTM, Tecan, Switzerland).

### Fluorescence-activated cell-sorting analysis for immune cell populations

Fluorescence-activated cell-sorting analysis for immune cell populations in the whole blood and spleen were analyzed using fluorescence-activated cell sorting (FACS). 100 μl of whole blood was collected using a whole blood collection tube (BD Vacutainer) and Fc receptor block was performed using anti-mouse CD16/CD32 Fc block antibody (BD Pharmingen) to reduce non-specific antibody binding for 3 min at room temperature. Then cells were incubated in the dark with 10 μl of the appropriate fluorochrome-conjugated antibodies from eBioscience-B cells (anti-CD19-APC, 1:100), Dedritic cell (anti-CD11C-PE, 1:25), NK cells (anti-CD49-APC, 1:50), T cells (anti-CD3-FITC, 1:400), and cytotoxic T cells (anti-CD8-FITC, 1:100) for 20 min at 4°C. Cells were washed with 500 μl of FACS buffer containing 0.02% sodium azide and 2% fetal bovine serum in PBS. The red blood cells were lysed for 5 minutes with FACS lysis buffer (BD Bioscience, Franklin Lakes, NJ, USA) at room temperature, then re-washed with FACS buffer. Each sample was finally fixed with 1% paraformaldehyde until further analysis. Flow cytometry was performed on the FACS Calibur system (BD Biosciences, Franklin Lakes, NJ, USA). Control samples were matched for each fluorochrome. Data were analyzed using CellQuest software (Becton Dickinson, Franklin Lakes, NJ, USA). Immune cell population analysis in the spleens and paw joint tissues were disrupted by forcing them through a 70-μm cell strainer into 10 ml of cold PBS by using a rubber-tipped syringe plunger. The cell suspensions were centrifuged at 1500 rpm for 10 min and the supernatants were discarded. The cells were resuspended in 3 ml ACK lysing buffer (LONZA, Walkersville, MD) for 3 minutes. The debris was sedimented by centrifuging at 1500 rpm for 10 minutes. Cell concentrations were determined by haemocytometer counting using trypan blue dye exclusion and were adjusted to 1×10^6^ cells/ml. Single-cell suspensions were stained with the following fluorochrome-conjugated antibodies from BD Biosciences: Dendritic cell (anti-CD11C,1:25), NK cells (anti-CD49-APC, 1:50). Flow cytometry and data analysis were done as mentioned above in blood.

### Statistical analysis

The data was analyzed using the GraphPad Prism 4 ver. 4.03 software (GraphPad Software, La Jolla, CA). Data is presented as mean ± SD. The homogeneity of variances was assessed using the Bartlett test. The differences in all data were evaluated by one-way analysis of variance (ANOVA). When the *P* value in the ANOVA test indicated statistical significance, the differences were evaluated by the Dunnett's test. The value of *P* < 0.05 was considered to be statistically significant.

## SUPPLEMENTARY FIGURES


