# Supplementary material for: Decreased severity of collagen antibody and lipopolysaccharide-induced arthritis in human IL-32β overexpressed transgenic mice
Source: Oncotarget. 2015 Oct 19;6(36):38566–77. doi: 10.18632/oncotarget.6160 (PMC4770721; doi:10.18632/oncotarget.6160)
Supplement: Supplementary file 1 [file oncotarget-06-38566-s001.pdf]

Decreased severity of collagen antibody and lipopolysaccharide-induced arthritis in human IL-32β overexpressed transgenic mice – Park et al

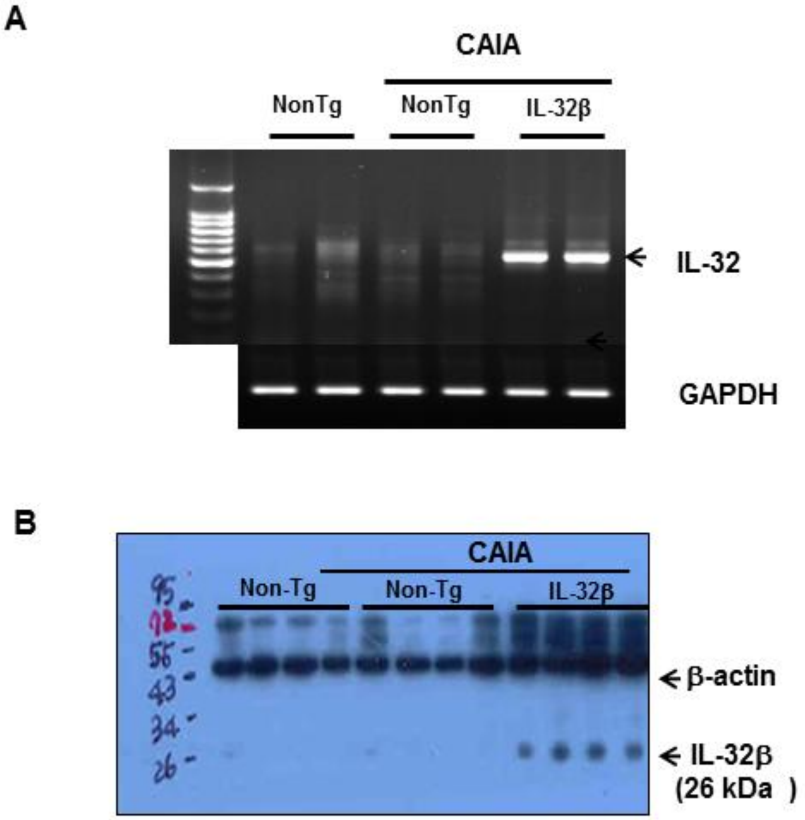

Supplementary Figure 1: The detection of IL-32β in IL-32β transgenic mice transgenic mice overexpressing human IL-32β generated with chicken beta-actin promoter was determined by RT-PCR (A) and Western blotting (B) in paw joint.

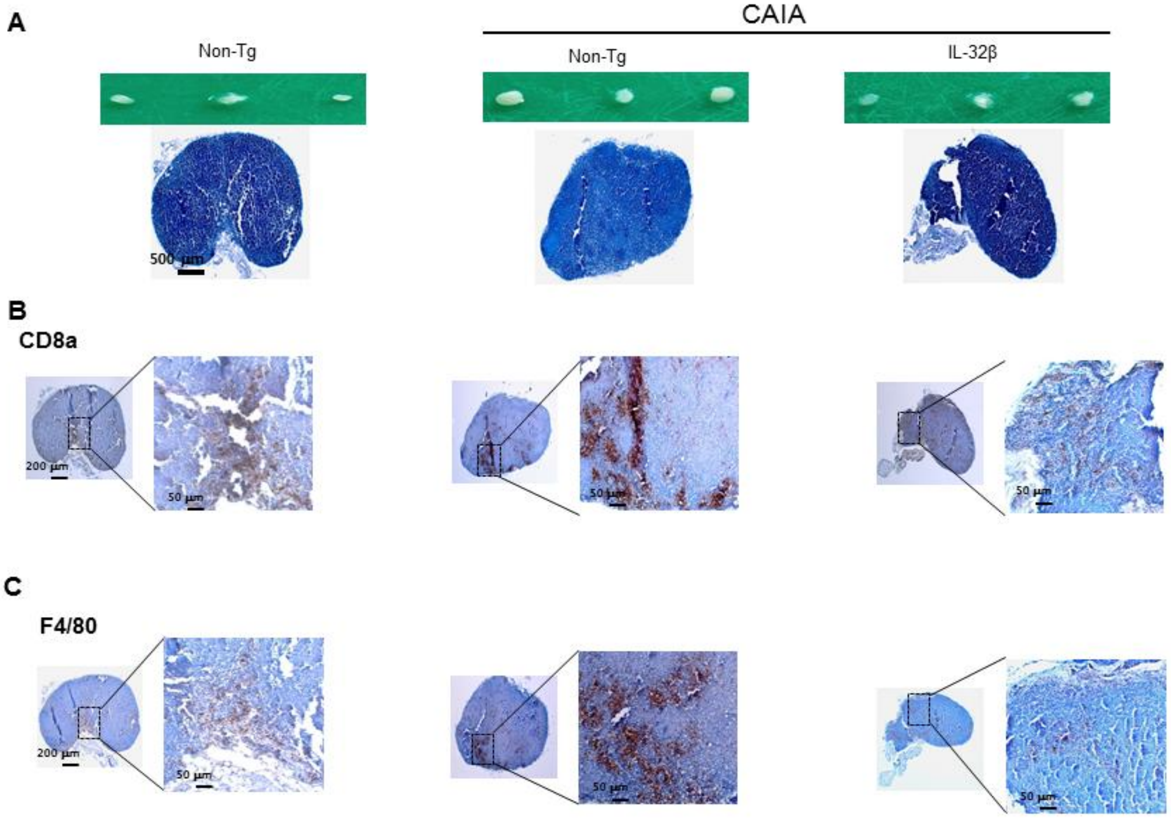

Supplementary Figure 2: The effect of IL-32β on lymph node and immune cells. (A) lymph node size were measured in CAIA-induced mice. (B-C) The expression level of CD8a and F4/80 were detected in the lymph node section.

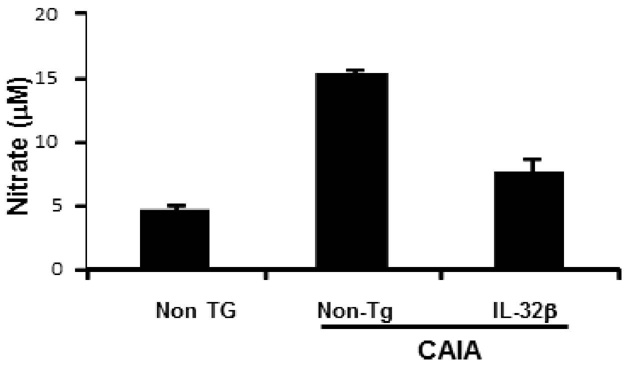

Supplementary Figure 3: The effect of IL-32β on cell proliferation and NO gereranion. 3H Thymidine incorporation and Nitrate were measured in paw joint tissues. *P<0.05 compared with non-Tg mice with CAIA. #P<0.05 compared without non-Tg mice with CAIA.
